# Iatrogenic Cushing Syndrome With Hypothalamic-Pituitary-Adrenal Axis Suppression in a Two-Month-Old Infant Following Unsupervised Topical Corticosteroid Use: A Case Report

**DOI:** 10.7759/cureus.117817

**Published:** 2026-10-12

**Authors:** Soukaina Bendouz, Hind Lachraf, Houda Bella, Zineb Imane, Asmaa Mdaghri Alaoui

**Affiliations:** 1 Neuropediatrics and Neurometabolic Diseases Unit, Children's Hospital of Rabat, Rabat, MAR; 2 Pediatric Diabetology and Endocrinology Unit, Children's Hospital of Rabat, Rabat, MAR; 3 Dysmorphology Unit, Children's Hospital of Rabat, Rabat, MAR

**Keywords:** adrenal insufficiency, betamethasone valerate, case report, diaper dermatitis, hypothalamic-pituitary-adrenal axis, iatrogenic cushing syndrome, infant, topical corticosteroids

## Abstract

Topical corticosteroids are commonly prescribed in pediatric dermatology; however, systemic adverse effects may occur rapidly in infants due to increased percutaneous absorption, the occlusive effect of diapers, and the immaturity of the hypothalamic-pituitary-adrenal (HPA) axis. We report the case of a two-month-old, 6 kg male infant who developed iatrogenic Cushing syndrome following approximately seven weeks of daily and repeated unsupervised application of a betamethasone valerate 0.1% topical corticosteroid to the diaper area for the treatment of diaper rash. Clinical examination revealed moon facies, a buffalo hump, and facio-truncal obesity. Laboratory evaluation showed suppressed morning cortisol at 5 µg/L (reference: 50-230 µg/L) and undetectable adrenocorticotropic hormone (ACTH) at 2.47 pg/mL (reference: 7-63 pg/mL), consistent with secondary adrenal insufficiency due to HPA axis suppression; brain CT showed no structural abnormality of clinical relevance. Management consisted of gradual withdrawal of the topical corticosteroid over four weeks, physiologic hydrocortisone replacement therapy at 8-10 mg/m²/day in three divided doses, and close clinical and biochemical follow-up. Biochemical recovery was suggested by normalization of morning cortisol to 272 µg/L, allowing progressive tapering and the discontinuation of hydrocortisone over six weeks without any episode of acute adrenal insufficiency. The family received structured education regarding the risks of unsupervised corticosteroid use and the warning signs of adrenal crisis. This case underscores that even moderate-to-potent topical corticosteroids applied to occluded skin areas can cause significant systemic toxicity in this vulnerable population and highlights the critical importance of parental education, appropriate prescribing guidance, and vigilant monitoring to prevent this avoidable complication.

## Introduction

Iatrogenic Cushing syndrome is the most common form of Cushing syndrome in childhood and results from exogenous glucocorticoid exposure via systemic, inhaled, or topical routes [1]. While topical corticosteroids are among the most widely prescribed agents in pediatric dermatology, systemic adverse effects may be underrecognized, particularly in infants, who are uniquely vulnerable due to their immature skin barrier, elevated body surface area-to-weight ratio, and the prolonged occlusive effect of diapers [1,2]. These factors significantly enhance percutaneous absorption, predisposing this population to rapid hypothalamic-pituitary-adrenal (HPA) axis suppression even with preparations whose systemic risk is underappreciated by caregivers. Prolonged or unsupervised exposure may therefore lead to HPA axis suppression, secondary adrenal insufficiency, and clinically overt Cushing syndrome, a complication that remains insufficiently recognized [2,3]. We report the case of a two-month-old infant who developed iatrogenic Cushing syndrome with biochemically confirmed HPA axis suppression following prolonged unsupervised application of a topical corticosteroid to the diaper area by the mother as self-medication for diaper rash.

## Case presentation

A two-month-and-20-day-old male infant with no significant medical history was referred for progressive facio-truncal obesity. Birth weight was 3.2 kg (50th percentile); weight at presentation was 6 kg (50th percentile), indicating that the infant was not overweight. History revealed repeated daily application of a potent topical corticosteroid (betamethasone valerate 0.1% cream, classified as Class III/potent according to the European classification) by the mother after each diaper change for approximately seven weeks, used as self-medication to treat diaper dermatitis, without medical prescription or supervision.

On physical examination, the infant presented with moon facies, buffalo hump, and an aspect of facio-truncal obesity due to abnormal fat redistribution. Height was 60 cm (50th percentile), and head circumference was 38.5 cm (25th percentile), both appropriate for age. Cutaneous examination showed moderate inflammatory diaper erythema without striae or ecchymosis. Psychomotor development was normal. These clinical findings are illustrated in Figure 1 and Figure 2.

**Figure 1:**
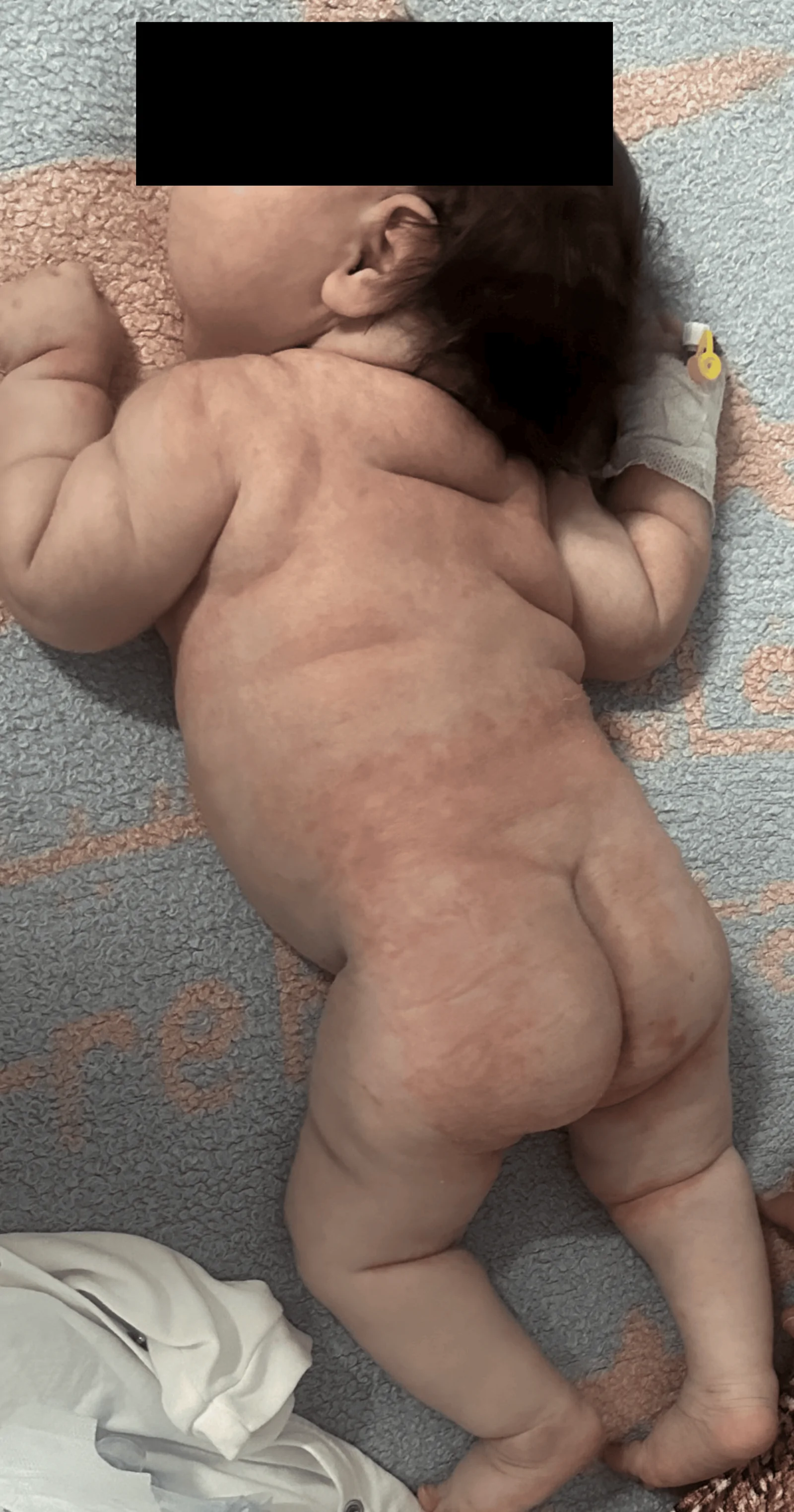
Posterior view demonstrating buffalo hump and truncal fat redistribution

**Figure 2:**
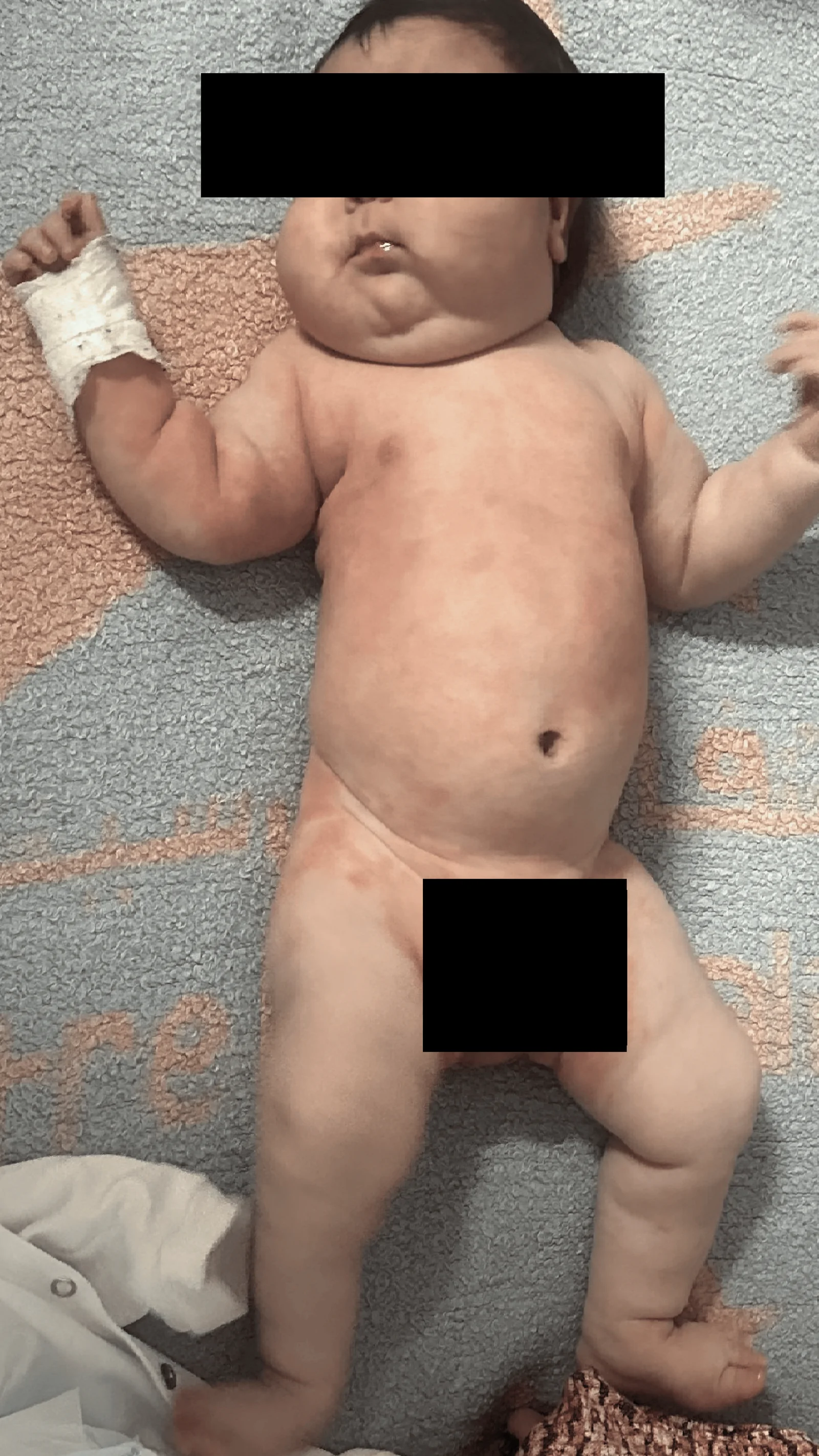
Anterior view demonstrating moon facies and truncal obesity

Laboratory investigations showed the following: sodium 137 mmol/L, potassium 5.1 mmol/L, fasting glucose 1.04 g/L, hemoglobin 11.7 g/dL, leukocyte count 6,240/mm³, and platelet count 661,000/mm³. Morning serum cortisol was suppressed at 5 µg/L (laboratory reference range: 50-230 µg/L), with a markedly suppressed adrenocorticotropic hormone (ACTH) level of 2.47 pg/mL (reference range: 7-63 pg/mL), supporting central HPA axis suppression. ACTH stimulation testing was not performed because synthetic ACTH (tetracosactide) was unavailable at our center at the time of evaluation. Given the characteristic Cushingoid phenotype, markedly suppressed morning cortisol, and low ACTH level in the setting of prolonged exogenous corticosteroid exposure, the patient was managed as having glucocorticoid-induced central adrenal suppression/secondary adrenal insufficiency. Brain CT was normal, with no structural pituitary or hypothalamic abnormality; therefore, the imaging is not illustrated because it did not provide clinically relevant additional information.

The topical corticosteroid was progressively withdrawn over four weeks with stepwise reduction in application frequency and quantity. Physiologic hydrocortisone replacement therapy was initiated at 8-10 mg/m²/day in three divided doses (approximately 3 mg/day for a body surface area of 0.32 m²). Clinical and biochemical monitoring was performed regularly. At the four-week follow-up, morning serum cortisol measured before the administration of the morning hydrocortisone dose had increased to 272 µg/L (Table 1). This finding was interpreted as supportive of biochemical recovery of HPA axis function; however, complete recovery could not be definitively confirmed because dynamic ACTH stimulation testing was not performed. Hydrocortisone was subsequently tapered over six weeks without any episode of adrenal crisis. Parental education regarding the signs and symptoms of adrenal insufficiency and the risks of unsupervised corticosteroid use was provided throughout follow-up. The clinical outcome was favorable, with marked improvement in the Cushingoid features and improvement in biochemical parameters.

**Table 1:** Biological parameters at admission and at four-week follow-up ACTH: adrenocorticotropic hormone

Parameter	At admission	At 4-week follow-up	Reference range
Morning cortisol	5 µg/L	272 µg/L	50-230 µg/L
ACTH	2.47 pg/mL	Not repeated	7-63 pg/mL
Sodium	137 mmol/L	135 mmol/L	135-145 mmol/L
Potassium	5.1 mmol/L	4.5 mmol/L	3.5-5.5 mmol/L
Fasting glucose	1.04 g/L	0.98 g/L	0.7-1.0 g/L

## Discussion

Systemic complications associated with topical corticosteroids in infants are uncommon but well documented [1]. Percutaneous absorption is influenced by several factors, including corticosteroid potency, duration of exposure, frequency of application, treated body surface area, and the degree of skin occlusion [1,2]. The diaper area represents a particularly high-risk site because humidity, skin maceration, and continuous occlusion can enhance transdermal glucocorticoid penetration. In our patient, repeated application of betamethasone valerate 0.1% cream to the diaper area after each diaper change for approximately seven weeks, without medical prescription or supervision, likely contributed to significant systemic absorption and HPA axis suppression [1,2]. To our knowledge, the age at presentation (two months) represents among the youngest cases reported in the literature of topical corticosteroid-induced iatrogenic Cushing syndrome, underscoring the particular vulnerability of neonates and young infants to this complication.

Exogenous glucocorticoids suppress hypothalamic corticotropin-releasing hormone (CRH) and pituitary ACTH secretion through negative feedback, potentially leading to secondary adrenal cortical atrophy and adrenal insufficiency [2-4]. In our patient, the presence of moon facies, buffalo hump, truncal obesity, markedly decreased morning cortisol (5 µg/L), and suppressed ACTH (2.47 pg/mL) was consistent with iatrogenic Cushing syndrome and central HPA axis suppression [2,3]. The mildly elevated platelet count (661,000/mm³) was considered a nonspecific finding and was interpreted cautiously [1]. Similar clinical and biochemical features have been reported by Ahmed et al. [5] in an infant exposed to a potent topical corticosteroid in the diaper area and by Kuikel et al. [6] in a pediatric patient with topical steroid overuse. Recent evidence also confirms that potent topical corticosteroids may induce significant HPA axis suppression in infants, particularly when applied to occluded skin areas [7]. These reports, together with our case, highlight the risk associated with prolonged and unsupervised use of potent topical corticosteroids, particularly on occluded skin.

The mild fasting hyperglycemia observed at admission (1.04 g/L; reference range: 0.7-1.0 g/L) may represent an additional metabolic effect of systemic glucocorticoid exposure [2,3]. It was clinically silent and normalized following corticosteroid withdrawal. Given its mild degree and nonspecific nature, it should not be considered a major diagnostic criterion for iatrogenic Cushing syndrome in this case.

Management consists of withdrawal of the offending corticosteroid combined with physiologic hydrocortisone replacement when significant HPA axis suppression is present, in order to prevent adrenal insufficiency during recovery [2,4]. In our patient, topical corticosteroid application was progressively reduced over four weeks and then discontinued, while physiologic hydrocortisone was administered at 8-10 mg/m²/day in three divided doses. Morning cortisol normalized to 272 µg/L after four weeks, and hydrocortisone was subsequently tapered over six additional weeks without any episode of adrenal crisis. This favorable outcome is consistent with the management approaches reported by Ahmed et al. [5] and Kuikel et al. [6], supporting corticosteroid withdrawal with temporary physiologic replacement in patients with clinically significant HPA axis suppression.

This case highlights the potential for significant systemic toxicity from potent topical corticosteroids in very young infants. Appropriate prescribing is therefore essential, particularly with regard to corticosteroid potency, duration, quantity, and application to occluded areas. Clear parental education regarding the risks of unsupervised corticosteroid use is also important. Clinicians should maintain a low threshold for evaluating HPA axis function when Cushingoid features develop in an infant with a history of prolonged or inappropriate topical corticosteroid exposure [8-12].

This case report has several limitations. First, a corticotropin stimulation test with tetracosactide (250 µg IV) could not be performed because synthetic ACTH was unavailable at our center at the time of evaluation. However, the combination of characteristic Cushingoid features, markedly suppressed morning cortisol, and undetectable ACTH was considered strongly supportive of HPA axis suppression and guided the decision to provide physiologic hydrocortisone replacement, consistent with previously reported cases [5,6]. Second, the exact commercial preparation and cumulative dose of betamethasone valerate could not be precisely quantified because the product was obtained without prescription and the amount used was reported retrospectively by the mother. Third, long-term follow-up beyond 10 weeks was unavailable. Although biochemical recovery was documented and hydrocortisone was successfully tapered, longer follow-up would have provided additional information regarding residual HPA axis vulnerability. Finally, as a single case report, the findings cannot be generalized to all infants receiving topical corticosteroids.

## Conclusions

Topical corticosteroids, particularly moderate- to high-potency preparations, can cause significant systemic adverse effects in young infants because of enhanced percutaneous absorption, especially when repeatedly applied to occluded areas such as the diaper region. This case highlights that prolonged unsupervised use may result in iatrogenic Cushing syndrome with marked HPA axis suppression and clinically significant glucocorticoid-induced adrenal insufficiency. Early recognition of Cushingoid features, appropriate endocrine evaluation, discontinuation of the offending corticosteroid, and adequate physiologic hydrocortisone replacement are essential to reduce the risk of adrenal insufficiency and adrenal crisis. In infants, topical corticosteroids should be used only under appropriate medical supervision, with careful attention to potency, treatment duration, quantity applied, and application site. Clinicians should provide caregivers with clear counseling regarding the potential risks of unsupervised corticosteroid use. Increased awareness of this rare but preventable complication may improve patient safety and reduce avoidable morbidity in this vulnerable population. In this case, clinical improvement and an increase in morning cortisol were suggestive of HPA axis recovery, although complete recovery could not be definitively established in the absence of dynamic ACTH stimulation testing and long-term follow-up.
